# Combination of 15 lipid metabolites and motilin to diagnose spleen-deficiency FD

**DOI:** 10.1186/s13020-019-0238-9

**Published:** 2019-04-15

**Authors:** Jiaqi Zhang, Xue Wang, Xiaoshuang Shi, Jingyi Xie, Min Zhang, Jinxin Ma, Fengyun Wang, Xudong Tang

**Affiliations:** 1 Department of Gastroenterology, Xiyuan Hospital of China Academy of Chinese Medical Sciences, No. 1, Xiyuan Caochang, Beijing, 100091 China; 2 Experimental Research Center of China Academy of Chinese Medical Sciences, Beijing, 100700 China; 3 Beijing University of Chinese Medicine, Beijing, 100029 China

**Keywords:** Functional dyspepsia, Spleen deficiency syndrome, Metabolomics, Biomarker

## Abstract

**Background:**

This study aims to assess clinical characteristics in FD with spleen deficiency syndrome and metabolic perturbations involved in FD progress. We combined metabolic biomarkers and clinical features into a better prediction for FD with Spleen Deficiency syndrome.

**Methods:**

A total of 276 people were recruited, including 215 FD patients and 61 healthy control group (HC). The clinical characteristics and gastric emptying rate were compared between spleen deficiency-FD group and non-spleen deficiency-FD. The serum lipids metabonomics analysis was performed to determine the metabolic differences in spleen deficiency-FD group and HC.

**Results:**

The symptoms of postprandial discomfort in Spleen Deficiency group were more severe (P < 0.05), and delayed gastric emptying was more pronounced (P < 0.05) vs. non-Spleen deficiency. Decreased motilin (OR = 0.990, 95% confidence interval (CI) 0.982–0.997) was independent risk factor related to Spleen Deficiency group. We identified 15 metabolites for spleen deficiency group vs. HC, majority of those biomarkers belonged to the glycerophospholipid metabolic pathway. The combination of 15 metabolics could diagnose spleen deficiency-FD, with the AUC of 0.9943, 95% CI 0.9854–1.0000), and the combination of 15 metabolics and motilin could diagnose spleen deficiency-FD, with the AUC of 0.9615, 95% CI 0.9264–9967).

**Conclusions:**

This study provides supportive evidence that Spleen deficiency syndrome was associated with delayed gastric emptying and the glycerophospholipid metabolic pathway was perturbed in FD patients. The combination of metabolic biomarkers and clinical features provided us with new ideas for multidimensional diagnosis of FD.

*Trial registration* http://www.chictr.org.cn, no: ChiCTR-TRC-13003200. clinicaltrials.gov, no: NCT02762136

**Electronic supplementary material:**

The online version of this article (10.1186/s13020-019-0238-9) contains supplementary material, which is available to authorized users.

## Background

Functional dyspepsia (FD) is a chronic disorder of the gastroduodenal region, which is one of the most prevalent functional gastrointestinal disorders. FD affects up to 15–20% of the worldwide population [1] and is associated with an important impact on quality of life and considerable healthcare expenses. The condition is diagnosed using the Rome III or IV criteria, which include the presence of epigastric pain or burning, early satiety during a normal-sized meal, or postprandial fullness, in the absence of an organic disease in the upper abdomen [2]. Traditional Chinese medicine considered that spleen deficiency syndrome is largely responsible for the pathogenesis of FD [3], especially postprandial discomfort syndrome (PDS).Spleen deficiency syndrome indicates that the organism is in a state of low energy metabolism and is particularly related to abnormal gastric motility [4, 5]. However, despite these findings, these conditions’ etiopathogenic mechanisms remain unclear, accounting for the lack of diagnostic biomarkers and the paucity of therapeutic options providing satisfactory long-standing clinical remission. An early and accurate diagnosis of FD is important in prognosis and early personalized medicine. At present, there is no specific examination method for the diagnosis of FD. The clinician primarily refers to the diagnostic criteria and excludes other organic diseases in the diagnosis. Previous studies have shown metabolomic changes in FD patients [6]. Given this background, we considered metabonomics to be an appropriate tool for determining the underlying variation of metabolites and to provide novel insight into the metabolism status of affected patients. Additionally, certain clinical characteristics related to FD with dysmotility, which can serve as risk factors for the occurrence of FD, may provide a supplement for the diagnosis of FD patients.

We conducted our study from two aspects: first, we profiled the clinical characteristics in order to investigate the clinical features related to FD with spleen deficiency syndrome; second, we performed a serum metabonomics analysis using ultra performance liquid chromatography quadrupole time-of-flight tandem mass spectrometry (UPLC-Q-TOF-MS/MS), investigating slight metabolic changes in the progress of FD and identifying specific biomarkers for FD with spleen deficiency syndrome. The combination of clinical features and metabolic biomarkers might facilitate clinical diagnosis for FD with spleen deficiency syndrome.

## Methods

The Additional file 1: Minimum Standards of Reporting Checklist contains details of the experimental design, and statistics, and resources used in this study. The overall workflow of serum metabonomics analysis utilized in this study is summarized in Additional file 2: Figure S1.

### Subjects

**Fig. 1:**
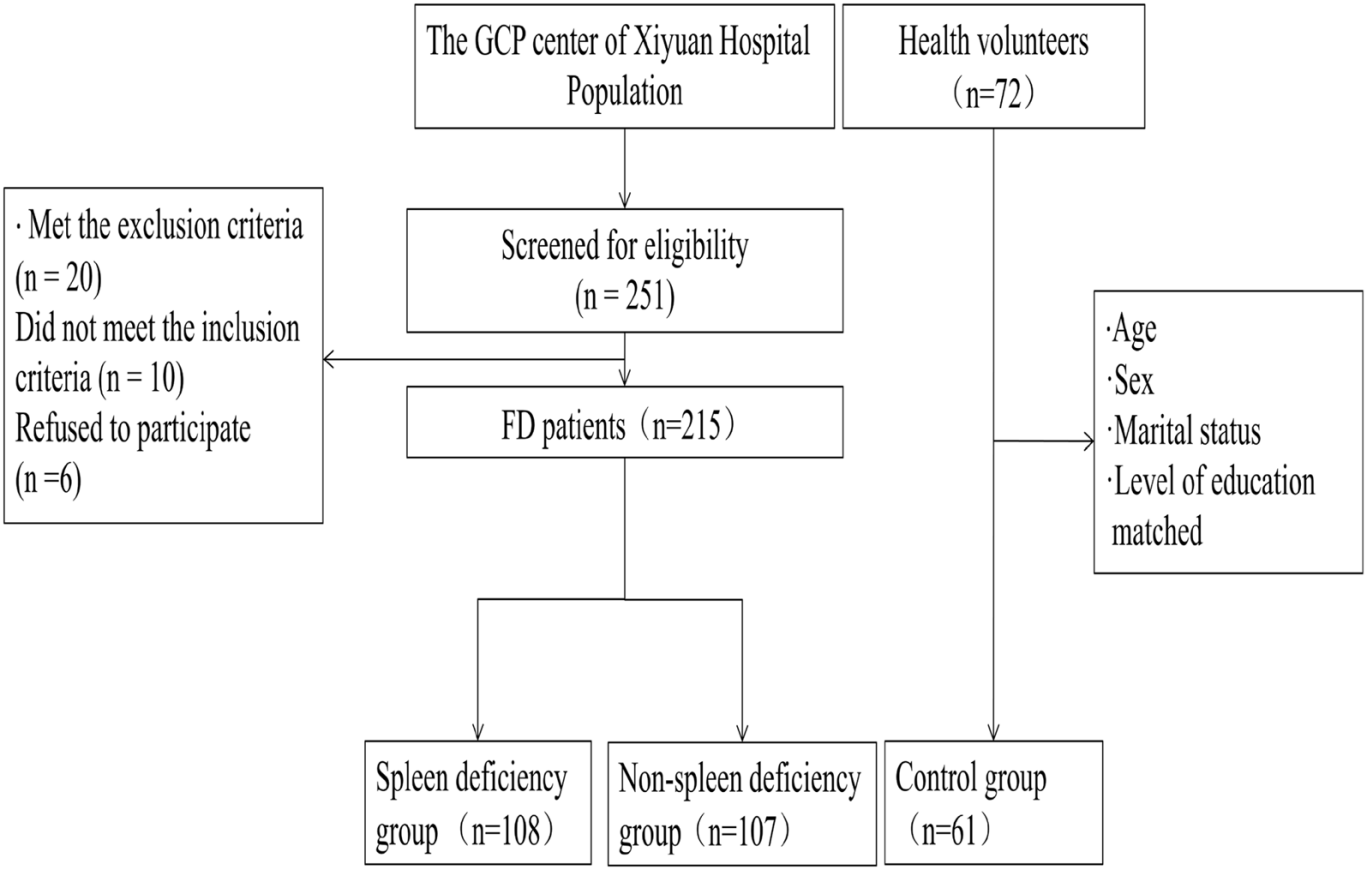
The flow chart of the selection process

This study was approved by the by the Ethical Review Committee of Xiyuan Hospital of China Academy of Chinese Medical Sciences (2013XL013-2) and conducted in accordance with the principles of the Declaration of Helsinki. All enrolled patients were confirmed to have signed informed consent after enrollment. Patients were recruited from the outpatients of three hospitals (XiYuan Hospital of China Academy of Chinese Medical Sciences, XY. GuangDong Province Traditional Chinese Medical Hospital, GD. WuHan Integrated TCM and Western Medicine Hospital, WH) in China between July 2013 and July 2016. Participants were recruited through advertising media, direct calls, and health promotion events. Advertisements were placed on notice boards and homepages of the hospitals and local newspapers. Patients were required to provide their medical history, receive a physical examination and laboratory safety tests, and undergo a gastroscopy. Only the patients who fulfilled the Rome III criteria (Additional file 2: Table S1) were considered eligible subjects. FD patients who met TCM Diagnostic Criteria for spleen deficiency syndrome (Additional file 2: Table S2) were included in the spleen deficiency syndrome group, and the rest were included in the non-spleen deficiency syndrome group. The inclusion and exclusion criteria are shown in Additional file 2: Table S3. Written informed consent was obtained from all patients prior to inclusion in the trial. Participants were free to withdraw from the study at any time. The healthy controls were made up of healthy volunteers with the same age recruited from the health examination center of the XiYuan hospital from May to July, 2016. All participants’ information were managed by the GCP center of XiYuan Hospital. The selection process used in this study is shown in Fig. 1.

### Clinical analysis

The patients’ epidemiological features, such as age of disease onset and sex, were collected. Also, clinical symptoms were recorded, such as postprandial fullness, early satiety, epigastric pain and epigastric burning according to the Postprandial Discomfort Severity Scale **(**Additional file 2: Table S4). All standard tests, including routine blood, urine and stool tests, pepsinogen, gastrin, ghrelin, motilin and cholecystokinin, were detected by enzyme-linked immune sorbent assays (ELISA). Continuous variables were described as the mean ± SD and compared by the independent sample t-test. Categorical variables were recorded as percentages and analyzed using the Chi square testy applying SPSS19.0.

### Gastric emptying (GE)

GE was observed using gastric ultrasound. Participants were asked to fast overnight before the ultrasonography. A 1120 mL esculent liquid was used as the test meal and was prepared by mixing 50 g of nutrient Cola Cao (enteral nutritional solid beverage, chocolate; Tianjin Cola Cao Food Co., Ltd., Tianjin, China) and 100 g of milk powder (Nestle whole milk powder; Shuangcheng Nestle Co., Ltd, Heilongjiang, China) with 1100 mL of warm water, and the entire test meal volume was 1120 mL. This esculent liquid contained 26.5 g of protein, 29.1 g of fat and 75.5 g of carbohydrate (840 kcal). This mixed liquid meal consisted of 13% proteins, 48% carbohydrates and 39% lipids and was used at a caloric density of 0.75 kcal/mL. The liquid test meal was administered orally as tolerated at a fixed rate of 50 mL/min, and all participants were allowed to sit on a chair for approximately 5 min while drinking this test meal. The subjects were scanned in a half-sitting position, sitting on an examining chair and leaning back at an angle of approximately 120°. Subjects were instructed not to move and to hold their breath at the end of expiration to permit diaphragmatic rising and restoration of the gastric configuration.

The proximal stomach and distal stomach volumes of each subject were scanned at six time points, which were fasting, maximum satiety, and 30 min, 60 min, 90 min, and 120 min after beginning ingestion.

The GE rate was calculated as follows: (Amax − A)/Amax × 100 (%), where Amax is the volume of maximum satiety after the meal in the proximal stomach or distal stomach, and A is the volume at other time points after the meal in the proximal stomach or distal stomach.

### Serum metabonomics analysis based on UPLCQ-TOF-MS/MS

#### Sample collection

Before the samples were collected, all participants were fasted for 12 h but were allowed to drink water to avoid the effects of food on the final outcome. Blood from both the healthy control group (n = 61) and spleen deficiency group (n = 90)were sampled via the antecubital vein, from which 5 mL was collected into heparinized test tubes and centrifuged at 3000*g* for 15 min at 4° C to separate the supernatant, and the supernatant was centrifuged at 3500*g* for 8 min at 4° C, and the supernatant obtained at the end served as the plasma sample. The plasma samples were collected and stored at − 80 °C for the metabonomic analysis.

#### Sample pretreatment

Plasma samples stored at − 80 °C were thawed at room temperature. To 150 *μ*L of plasma, 450 L of methanol was added, vortexed for 30 s and allowed to stand for 10 min. The mixture was centrifuged at 12,000 rpm, 4 °C for 10 min, and the supernatant was injected for UPLC-Q-TOF/MS analysis. In addition, 400 μL of the supernatant was dried with nitrogen, reconstituted in 75 μL of ultrapure water, vortexed for 30 s, allowed to stand for 10 min, and centrifuged at 12,000 rpm at 4 °C for 10 min, and 10 μL of the supernatant was placed in a vial containing an inner liner for later use.

#### Chromatographic and mass spectrometric conditions

##### Chromatographic conditions

WATERS ACQUITY UPLC I-CLASS Liquid System, Column HSS T3 Column (2.1 mm × 100 mm, 1.8 μm, Waters, UK), column temperature 40 °C, elution system 0.1% formic acid water (a): 0.1% formic acid acetonitrile (B), gradient elution program: 0 ~ 1 min, 1%B ~ 1%B; 1 ~ 2 min, 1B%–45%B; 2 ~ 5 min, 45B ~ 70%B; 5 ~ 9 min, 70%B ~ 90%B; 9 ~ 12 min, 90%B ~ 99%B; 12 ~ 15 min, 99%B ~ 99%B; 15–15.1 min, 99%B ~ 1%B; 15.1 ~ 17 min, 1%B ~ 1%B. The sample chamber temperature was 4 °C, the injection volume was 2.0 μL, and the flow rate was 0.30 mL min^−1^.

##### Mass spectrometric conditions

SYNAPT G2-SI Q/TOF tandem quadrupole time-of-flight mass spectrometry system used ESI positive and negative ion mode detection. The accurate mass was determined with leucine-enkephalin. The scanning range was 50–1200 Da. The data acquisition mode was 3D data acquisition in continuum mode. Low energy channel collision voltage: 6 V, high energy channel collision voltage: 10–40 V. In positive ion mode, capillary (kV): 3.0, sampling cone: 30 V, source offset: 80, source temperature: 100 °C, desolvation temperature: 400 °C, cone gas: 40 (L/h), desolvation gas flow (L/h): 800, nebulizer (bar): 7.0. In negative ion mode, capillary (kV): 2.2, other parameters are the same as the parameters in positive ion mode.

#### Data processing and statistical analysis

UPLC-Q-TOF/MS raw data were analyzed using Progenesis QI software. The software performed baseline calibration, peak alignment, peak identification, feature peak extraction, and normalization operations. The raw data were converted into a three-dimensional matrix containing tR-m/z ion pairs, sample names, and peak intensities. The acquired data were imported into multivariate statistical software EZinfo 2.0 for multivariate data analysis (PCA, OPLS-DA and S-plot). Metabolite with variable importance plot (VIP) > 1 was screened as a potential biomarker. An independent sample -test was later performed using SPSS 17.0 software to determine the statistical significance of metabolites. By comparison with authentic standards, MS/MS fragmentation, and HMDB, the significantly varied biomarkers were identified. Next, the biomarkers were optimized using the receiver operating characteristic (ROC) curve by SPSS. Finally, MetPA (https://www.metaboanalyst.ca/) was utilized to describe the involved metabolic pathways of the biomarkers.

## Results

### Characteristics of patients

**Fig. 2:**
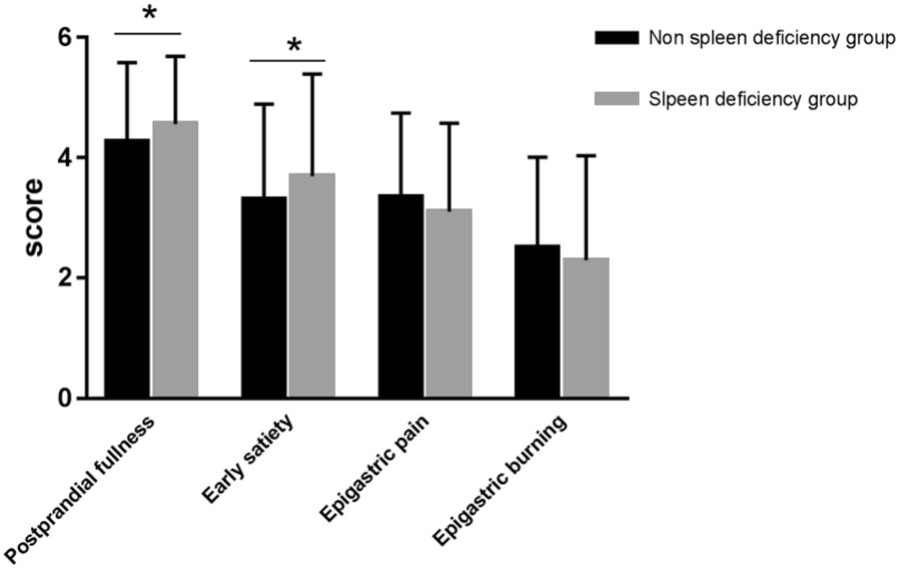
Postprandial discomfort severity scale (*p < 0.05, **p < 0.01 vs. Non spleen deficiency-FD group). The spleen deficiency group had more severe postprandial fullness and early satiety symptoms compared with the non-spleen deficiency group (p < 0.05)

Clinical characteristics were compared among the spleen deficiency group, non-spleen deficiency group and healthy control group (Table 1). There was no significant difference in gender distribution, age, marital status, level of education or body mass index (BMI) among the three groups. Postprandial discomfort severity scale **s**howed that the spleen deficiency group had more severe postprandial fullness (4.56 ± 1.13 [spleen deficiency-FD group] vs. 4.27 ± 1.31 [Non spleen deficiency group], *p *< 0.05) and early satiety (3.69 ± 1.70 vs. 3.31 ± 1.58, *p *< 0.05) symptoms compared with the non-spleen deficiency group (Table 1 and Fig. 2).

**Table 1:** Baseline characteristics of enrolled patients

Parameter	Spleen deficiency group (n = 108)	Non spleen deficiency group (n = 107)	Healthy control group (n = 61)
Gender			
Male	34 (31.48%)	42 (39. 25%)	20 (32.79%)
Female	74 (68.52%)	65 (60.75%)	41 (67.21%)
Age	46.98 ± 12.74	46.93 ± 12.91	25.90 ± 2.89
Marital status			
Unmarried	21 (19.44%)	20 (18.69%)	12 (16.67%)
Married	86 (79.63%)	83 (77.57%)	47 (77.05%)
Divorced	1 (0.93%)	4 (3.74%)	4 (3.28%)
Widowhood	0 (0%)	0 (0%)	0 (0%)
Level of education			
Illiteracy	3 (2.78%)	1 (0.94%)	1 (1.64%)
Primary school	5 (4.63%)	6 (5.61%)	4 (6.56%)
Junior school	19 (17.59%)	14 (13.08%)	12 (19.67%)
Senior school	17 (15.74%)	25 (23.36%)	20 (32.79%)
Degree or above	64 (59.26%)	61 (57.01%)	24 (39.34%)
Body mass index (BMI)	21.82 ± 3.17	21.97 ± 3.05	22.16 ± 2.04
Postprandial discomfort severity scale			
Postprandial fullness	4.56 ± 1.13*	4.27 ± 1.31	–
Early satiety	3.69 ± 1.70*	3.31 ± 1.58	–
Epigastric pain	3.10 ± 1.47	3.35 ± 1.39	–
Epigastric burning	2.29 ± 1.74	2.51 ± 1.50	–

* *p *< 0.05

**Fig. 3:**
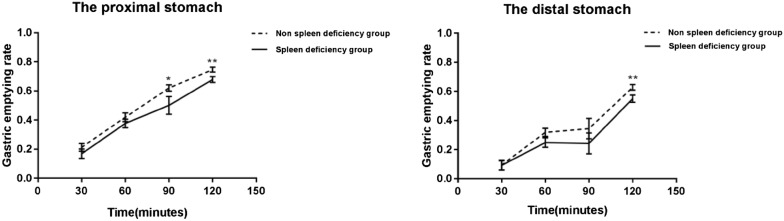
Gastric emptying rates of proximal and distal stomach (*p < 0.05, **p < 0.01 vs. Non spleen deficiency group). The gastric emptying rate of the proximal stomach (GERPG) in the spleen deficiency group significantly decreased at 90 min (p < 0.05) and 120 min (p < 0.01); the gastric emptying rate of the distal stomach (GERDG) in the spleen deficiency group decreased at 120 min (p < 0.01)

The gastric emptying rate in the spleen deficiency group was generally lower than that in the non-spleen group. Compared with the non-spleen deficiency group, the gastric emptying rate of the proximal stomach (GERPG) in the spleen deficiency group significantly decreased at 90 min (*p *< 0.05) and 120 min (*p *< 0.01); moreover, the gastric emptying rate of the distal stomach (GERDG) in the spleen deficiency group decreased at 120 min (*p *< 0.01).The results indicated that gastric emptying occurred more frequently in FD with spleen deficiency (Fig. 3).

**Fig. 4:**
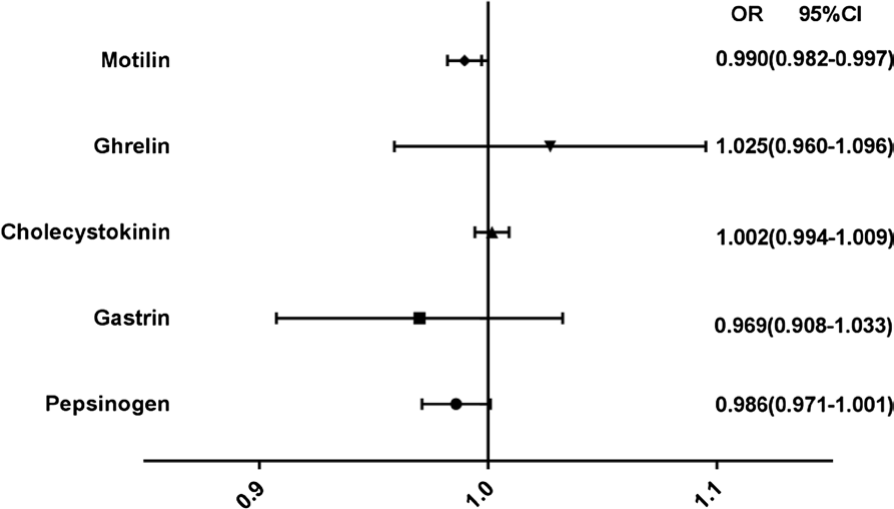
Risk factors for the FD patients with spleen deficiency syndrome. The results of multivariate logistic regression shown that decreased motilin was an independent risk factor related to spleen deficiency in patients with FD

We examined hormone levels related to digestive function and gastrointestinal motility. Compared with the healthy control group, the level of pepsinogen (256.53 ± 69.53 [spleen deficiency-FD group] vs. 310.00 ± 66.06 [healthy control group], *p *< *0.01*), gastrin (64.91 ± 17.52 vs. 85.82 ± 11.41, *p *< *0.01*), ghrelin (75.58 ± 17.52 vs. 98.67 ± 12.92, *p *< *0.01*), motilin (555.85 ± 128.12 vs. 754.01 ± 113.27, *p *< *0.01*) were significantly reduced and the level of cholecystokinin (688.96 ± 114.35 vs. 647.71 ± 93.64, *p *< *0.05)* was increased in the spleen deficiency group. Further studies showed that compared with the non-spleen deficiency group, the level of pepsinogen (256.53 ± 69.53 [spleen deficiency-FD group] vs. 314.18 ± 55.91 [non spleen deficiency-FD group], *p *< *0.01*), gastrin (64.91 ± 17.52 vs. 80.88 ± 12.32, *p *< *0.01*), motilin (555.85 ± 128.12 vs. 709.39 ± 135.08, *p *< *0.01*) were significantly reduced and the level of cholecystokinin (688.96 ± 114.35 vs. 630.76 ± 77.83, *p *< 0.05) was increased in the spleen deficiency group. No significant difference was found between the spleen deficiency-FD group and the non-spleen deficiency-FD group regarding the level of ghrelin (*p *= 0.065 > 0.05) (Table 2). Additionally, the results of multivariate logistic regression shown that decreased motilin was an independent risk factor related to spleen deficiency in patients with FD (Fig. 4).

**Table 2:** Laboratory data of enrolled patients

Group	Pepsinogen (ng/mL)	Gastrin (ng/mL)	Cholecystokinin (pg/mL)	Ghrelin (ng/mL)	Motilin (pg/mL)
Spleen deficiency	256.53 ± 69.53††**	64.91 ± 17.52††**	688.96 ± 114.35†*	75.58 ± 17.52††	555.85 ± 128.12††**
Non-spleen deficiency	314.18 ± 55.91	80.88 ± 12.32	630.76 ± 77.83	83.51 ± 11.32	709.39 ± 135.08
Healthy control	310.00 ± 66.06	85.82 ± 11.41	647.71 ± 93.64	98.67 ± 12.92	754.01 ± 113.27

* *p *< 0.05, ** *p *< 0.01 vs. Non spleen deficiency-FD group; † *p *< 0.05, †† *p *< 0.01 vs. Healthy control group

### Metabolic profiles of spleen deficiency-FD group and healthy control group

**Fig. 5:**
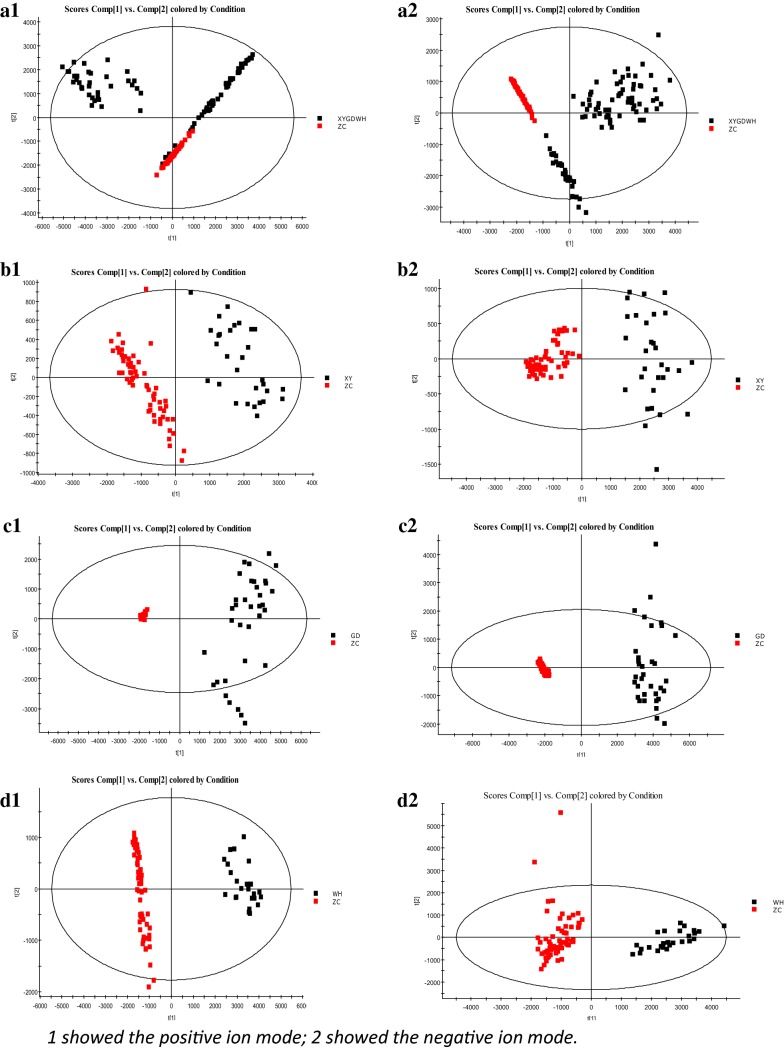
The PCA scores of spleen deficiency-FD group and healthy control group. (**a** All spleen deficiency-FD patients and healthy control group; **b** spleen deficiency-FD patients of XiYuan Hospital and healthy control group; **c** spleen deficiency-FD patients of GuangDong Province Traditional Chinese Medical Hospital and healthy control group; **d** spleen deficiency-FD patients of WuHan Integrated TCM &Western Medicine Hospital and healthy control group)

Figure 5 shows the principal component analysis (PCA) scores of the spleen deficiency-FD group (under positive and negative ion patterns). However, the spleen deficiency group and healthy control group did not have a poor clustering trend. The patients in the three hospitals could not be well-grouped, and the FD patients from the three hospitals could not be well-grouped (Fig. 5a1, a2). The reasons for this might be (1) the three hospitals used different sample collection methods; (2) there were certain differences in patients’ physiques in the different regions; or (3) ambient conditions during sample preservation or transportation influenced the final results. Therefore, we were motivated to engage in statistical analysis of spleen deficiency-FD patients in the three hospitals for biomarkers that could characterize FD. The results showed that the spleen deficiency-FD group was completely separated from the healthy control group (Fig. 5b–d).

### Identification of biomarkers

**Fig. 6:**
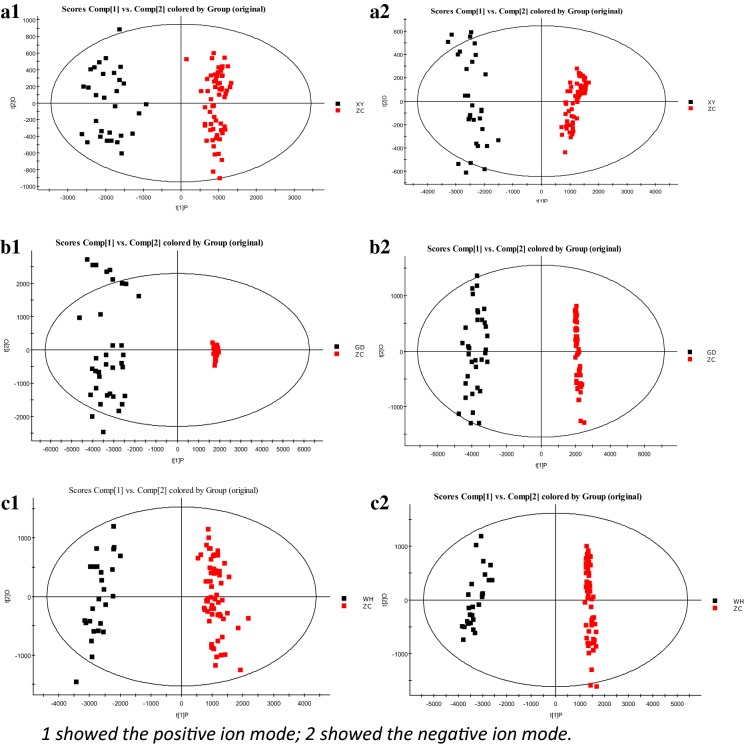
The OPLS-DA scores of spleen deficiency-FD group and healthy control group. (**a** spleen deficiency-FD patients of XiYuan Hospital and healthy control group; **b** spleen deficiency-FD patients of GuangDong Province Traditional Chinese Medical Hospital and healthy control group; **c** spleen deficiency-FD patients of WuHan Integrated TCM &Western Medicine Hospital and healthy control group)

**Fig. 7:**
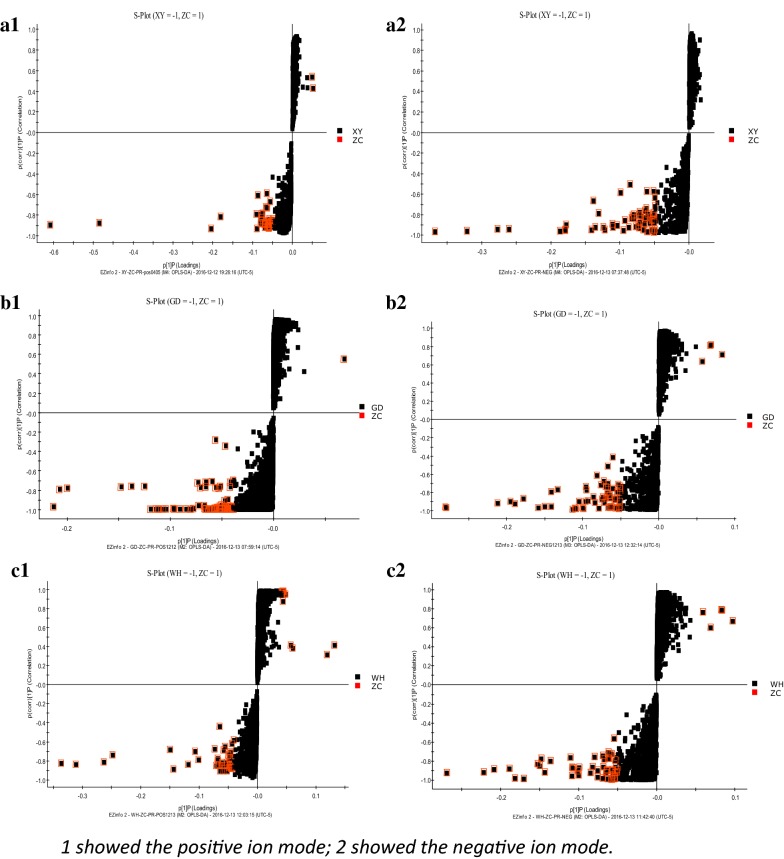
The S-Plot of spleen deficiency-FD group and healthy control group. (**a** spleen deficiency-FD patients of XiYuan Hospital and healthy control group; **b** spleen deficiency-FD patients of GuangDong Province Traditional Chinese Medical Hospital and healthy control group; **c** spleen deficiency-FD patients of WuHan Integrated TCM &Western Medicine Hospital and healthy control group)

**Fig. 8:**
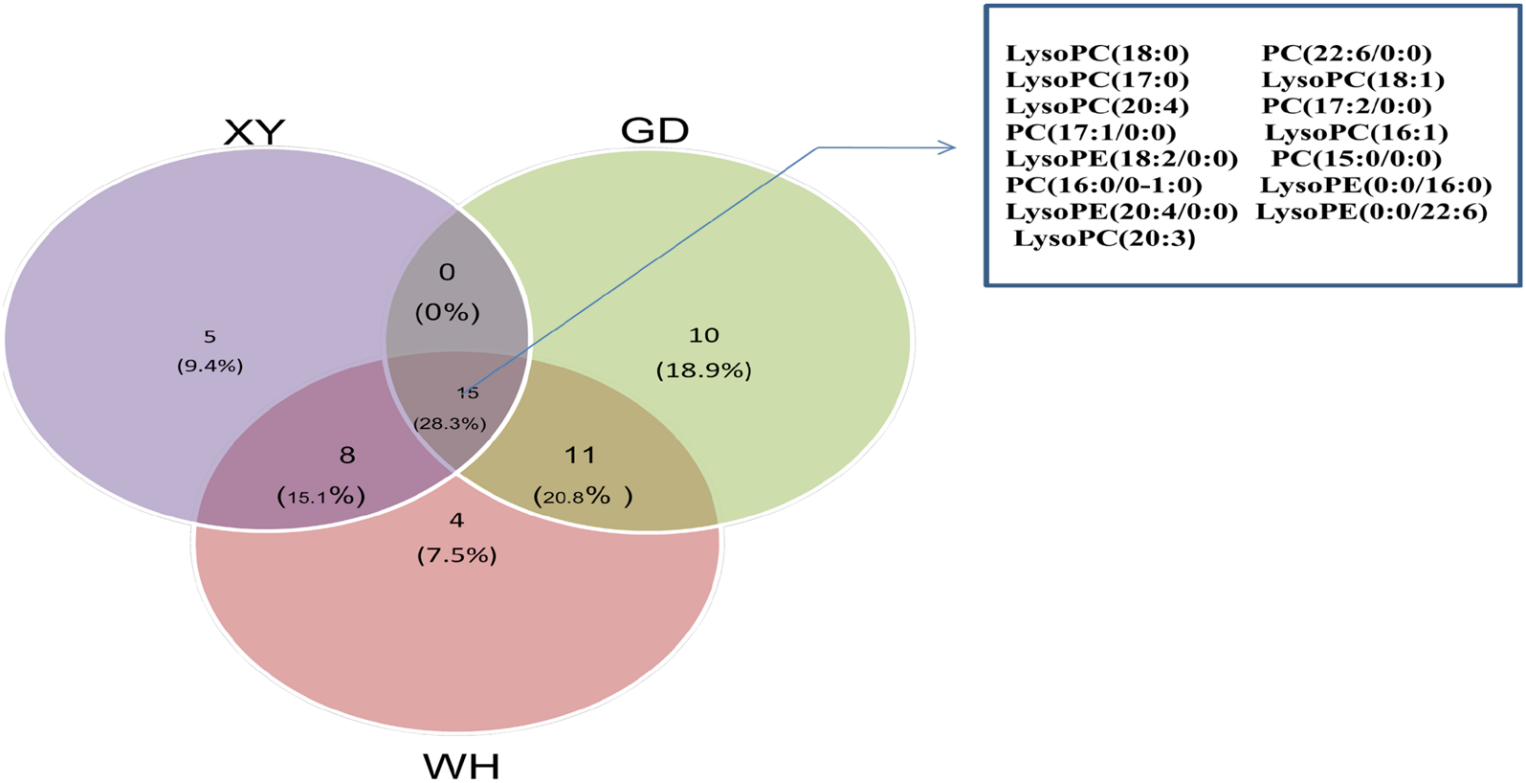
Venn plot for all metabonomics biomarkers among three groups. There are 15 overlapping metabolites: LysoPC (18:0), PC (22:6/0:0), LysoPC (17:0), LysoPC (18:1), LysoPC (20:4), PC (17:2/0:0), PC (17:1/0:0), LysoPC (16:1), LysoPE (18:2/0:0), PC (15:0/0:0), PC (16:0/0-1:0), LysoPE (0:0/16:0), LysoPE (20:4/0:0), LysoPE (0:0/22:6), LysoPC (20:3)

**Fig. 9:**
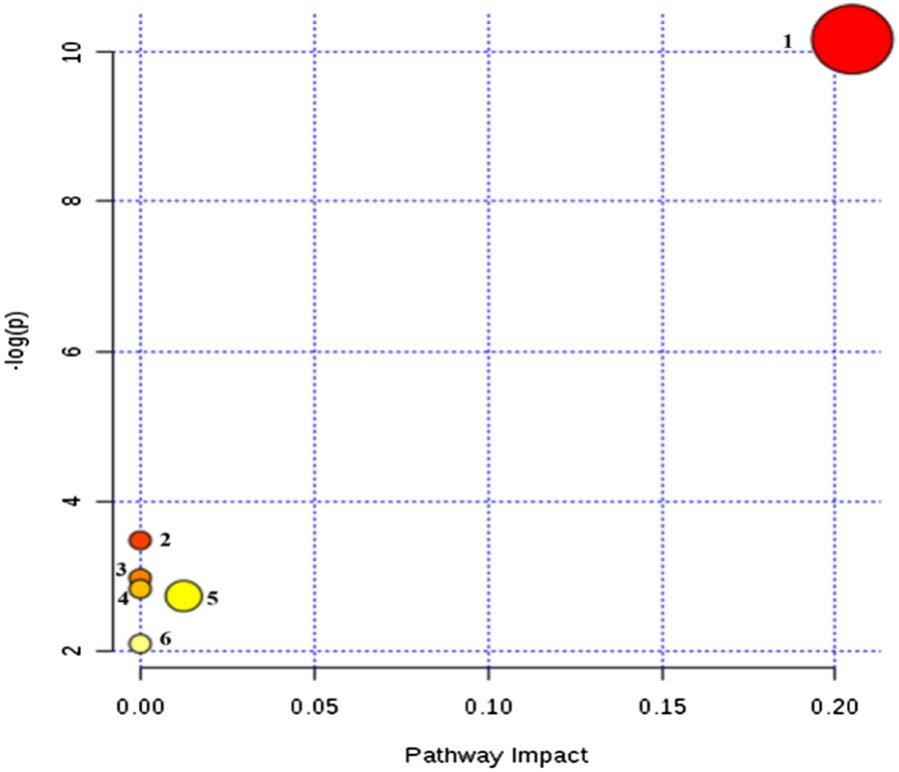
Metabolism map of FD with spleen deficiency syndrome. (1) glycerol phospholipid metabolism; (2) linoleic acid metabolism; (3) sphingolipid metabolism; (4) alpha-linolenic acid metabolism; (5) glyceride metabolism; (6) arachidonic acid metabolism

In order to identify different metabolites between the spleen deficiency-FD group of each hospital and the healthy control group, orthogonal single collection partial least discriminant analysis (OPLS-DA) was performed (Fig. 6). Figure 7 shows the S-Plot results of the spleen deficiency-FD group and the healthy control group. The red box in the figures shows the important differential metabolites selected in the small molecule metabolome according to the screening conditions VIP > 1, p < 0.05, and FC value > 2. The differential metabolites were identified by accurate mass number search, MS/MS data inference obtained from MSE data, and online database lysis information comparison(Additional file 2: Tables S5, S6, S7). There were 29 potential biomarkers identified in the Xiyuan Hospital Group, 37 in the Guangdong Hospital Group and 40 in the Wuhan Hospital Group. Most of the biomarkers were found to be PC (phosphatidylcholine), PE (phosphatidylethanolamine), PA (phosphatidic acid), and PS (phosphatidylserine) (Additional file 2: Tables S4, S5, S6). The Venn plot depicted the overlap of biomarkers for the three spleen deficiency-FD groups as shown in Fig. 8. Metabolic pathways were determined for potential biomarkers using the MetPA website, and the majority of these 15 metabolites biomarkers belonged to the glycerophospholipid metabolic pathway (Fig. 9).

The ROC presentations for spleen deficiency-FD vs. HC appear in Table 3. The prediction of spleen deficiency-FD vs. HC was robust, and the area under the curve (AUC) of 15 biomarkers for spleen deficiency-FD vs. HC ranged from 0.6790 to 0.9926. The combination of 15 biomarkers could offer a highly accurate diagnosis of spleen deficiency in FD with an AUC of 0.9943 (Table 3 and Fig. 9).

**Table 3:** Results of AUCs for biomarkers

Biomarkers and laboratory data	Spleen deficiency-FD vs. HC		
	AUC	P-value	95% CI
Top biomarkers			
LysoPC (18:0)	0.8053	< 0.0001	–
PC (22:6/0:0)	0.9039	< 0.0001	–
LysoPC (17:0)	0.6790	0.0002	–
LysoPC (18:1)	0.9895	< 0.0001	–
LysoPC (20:4)	0.9793	< 0.0001	–
PC (17:2/0:0)	0.8891	< 0.0001	–
PC (17:1/0:0)	0.9926	< 0.0001	–
LysoPC (16:1)	0.9441	< 0.0001	–
LysoPE (18:2/0:0)	0.9438	< 0.0001	–
PC (15:0/0:0)	0.9655	< 0.0001	–
PC (16:0/0–1:0)	0.9267	< 0.0001	–
LysoPE (0:0/16:0)	0.7155	< 0.0001	–
LysoPE (20:4/0:0)	0.9633	< 0.0001	–
LysoPE (0:0/22:6)	0.9743	< 0.0001	–
LysoPC (20:3)	0.9136	< 0.0001	–
Laboratory data			
Motilin	0.8487	< 0.0001	–
The combination of top biomarkers	0.9943	< 0.0001	0.9845–1.000
The combination of top biomarkers and motilin	0.9615	< 0.0001	0.9264–0.9967

### Data integration to improve prediction

**Fig. 10:**
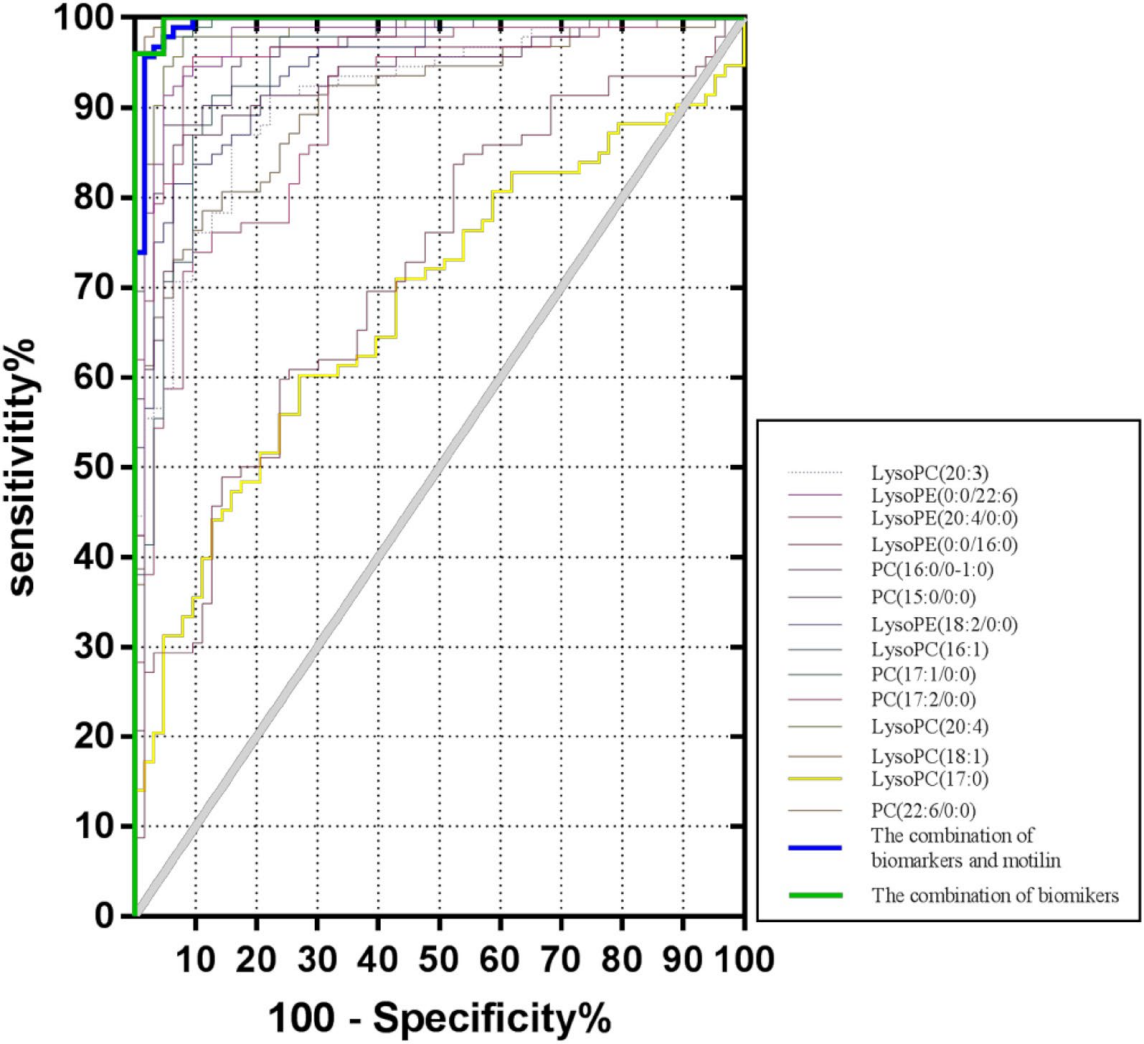
ROC curves of biomarkers for Spleen-deficiency in prediction. The prediction of spleen deficiency-FD vs. HC was robust, and the area under the curve (AUC) of 15 biomarkers for spleen deficiency-FD vs. HC ranged from 0.6790 to 0.9926. The combination of 15 biomarkers could offer a highly accurate diagnosis of spleen deficiency in FD with an AUC of 0.9943

Metabonomics data were integrated with clinical data to improve the prediction for spleen deficiency in FD. Results of multivariate logistic regression analyses are shown in Table 3. All features were significant after multivariate adjustment. Although the integrated data did not strengthen the prediction capacity for spleen deficiency-FD, the combination of biomarkers and decreased motilin provided excellent diagnostic capabilities with an AUC of 0.9615 (Table 3 and Fig. 10).

## Discussion

Large-scale studies reported a 10–30% prevalence of FD worldwide [7]. The pathophysiology of FD is likely complex and multifactorial and has not been thoroughly elucidated to date. Gastroduodenal motor and sensory dysfunction, as well as impaired mucosal integrity, low-grade immune activation, and dysregulation of the gutbrain axis, have all been implicated [8]. At present, the pathophysiology of functional dyspepsia is only partially elucidated. There is growing evidence that functional dyspepsia is a highly heterogeneous disorder, and different subgroups can be identified based on different demographic, clinical, and pathophysiologic features. However, there is still a lack of objective biomarkers for the diagnosis and evaluation of FD.

In the present study, a total of 215 FD patients with or without spleen deficiency syndrome and 61 healthy people were enrolled. According to our data, the spleen deficiency group had more severe postprandial fullness and early satiety symptoms, and delayed gastric emptying occurred more often in the spleen deficiency group than in the non-spleen deficiency group. The rate of proximal gastric emptying at 90 and 120 min and the distal gastric emptying rate at 120 min were significantly lower than those of the non-spleen deficiency group (P < 0.05).Because delayed gastric emptying is considered a pathophysiological mechanism of functional dyspepsia [9–11], some experts advocate the assessment of this parameter in the diagnostic work-up. However, the correlation between gastric emptying and dyspeptic symptoms is still unclear and treatment of delayed gastric emptying with prokinetic drugs poorly correlates with symptomatic improvement [12]. Gastric emptying is delayed in a sizable fraction (estimates vary from about 25% to 35%) of unselected FD patients, while rapid gastric emptying is uncommon, probably occurring in under 5% of cases [13, 14]. Moreover, gastric emptying measurements are expensive and not widely available as a well-standardized test. Quantification of gastric emptying is therefore not advocated in the standard clinical management of functional dyspepsia [15]. Gastric emptying could be assessed by ultrasonography, but this approach requires expertise and is time-consuming; thus, it should be considered to be a research tool only [11]. Spleen deficiency syndrome is a pathological condition of multiple system dysfunction, characterized by gastrointestinal digestion hypofunction and a gastrointestinal motility disorder [16]. Epidemiological studies show that the incidence of spleen deficiency syndrome in functional dyspepsia is 64.04% [17]. Interestingly, many studies have shown that there was a significant positive correlation between spleen deficiency syndrome and delayed gastric emptying rate [18–21] in line with our research results. Traditional Chinese medicine diagnoses of spleen deficiency syndrome are based on clinical symptoms combined with tongue coating and pulse, which provides a new strategy for the diagnosis of delayed gastric emptying rate in functional dyspepsia.

Recently, evidence has accumulated that delayed gastric emptying rate is closely associated with altered gastrointestinal hormones [22, 23] in the current study, we showed that gastrointestinal hormones (pepsinogen, gastrin, ghrelin, motilin) were significantly reduced, and the cholecystokinin level was increased, in the spleen deficiency-FD group compared with the non-spleen deficiency-FD group and healthy control group. Gastrin and pepsinogens are representative biomarkers that influence the gastric physiology and thus reflect the functional state of the gastric mucosa [24]. Gastrin stimulates gastric acid secretion and mucosal cell growth. Pepsinogen (PG) I is secreted in the mucosa of the gastric corpus. PG II is secreted not only in the corpus but also in the antrum. Since PGs are secreted by chief cells in the gastric mucosa, their serum levels may reflect the mass and/or turnover of those cells in the mucosa [25]. It has been reported that measuring these markers in the serum thus allows gastric pathologies, such as atrophic gastritis, FD and abnormalities in acid secretion, to be detected [26, 27]. Cholecystokinin binds to receptors on vagal afferents and mucosal receptors in the stomach and small intestine to potentiate gastric relaxation, stimulate mechanoreceptors sensitive to gastricstretch, and slow gastric emptying [28]. Motilin is a hormone released by the endocrine cells of the duodenal mucosa during fasting to stimulate gastrointestinal motility [29]. Ghrelin, the closest family member of motilin, has now emerged as a multifunctional hormone with important effects on energy homeostasis but also on gastrointestinal motility; similar to motilin, it induces hunger contractions in the fasting state and acts postprandially to accelerate gastric emptying. Our findings are consistent with most studies, suggested that there were more serious digestive disorders in the spleen deficiency-FD group.

Most studies have shown that motilin and ghrelin accelerate gastric emptying and cholecystokinin slows gastric emptying. In our study, multivariate logistic regression showed that only decreased motilin was an independent risk factor related to spleen deficiency in patients with FD. Our findings confirm and extend the connection between spleen deficiency syndrome and a delayed gastric emptying rate.

Metabolic profiling revealed significant metabolic differences between the spleen deficiency-FD group and the healthy control group. The OPLS-DA score plots of all samples displayed clear separations for the spleen deficiency-FD group vs. HC at the three centers, suggesting that the spleen deficiency-FD group had a common series of metabolic changes at all three centers. A total of 15 overlapping potential biomarkers were identified through Venn plots. Further research showed that most of them were found to be PC (phosphatidylcholine), PE (phosphatidylethanolamine), PA (phosphatidic acid), and PS (phosphatidylserine).Metabolic pathways were determined for potential biomarkers using the MetPA website, and the majority of these 15 metabolites biomarkers belonged to the glycerophospholipid metabolic pathway. The AUC of 15 biomarkers for spleen deficiency-FD vs. HC ranged from 0.6790 to 0.9926, and the combination of 15 metabolics strengthened the prediction capacity for spleen deficiency-FD with an AUC of 0.9943, suggesting that these 15 metabolites as biomarkers were robust. Although there was no increase in the prediction capacity for spleen deficiency-FD, the combination of metabolic biomarkers and motilin provided us with new ideas for multidimensional diagnosis of FD.

Regarding the results of lipid metabolism, our findings are consistent with Wu Qiaofeng’S [30]. In her research, besides abnormal phospholipid metabolism, metabolic changes in FD patients included increased glucose degradation, increased fat mobilization, increased gluconeogenesis, and reduced leucine/isoleucine levels. Disturbance of glucose and lipid metabolism in FD patients suggested that the rate of glycolysis is reduced in these patients, and energy consumption is switched to lipid oxidation. Recent evidence indicates that as a group, FD patients eat fewer meals and consume less energy compared to healthy individuals [31], and another study showed that high-fat meals could induce FD symptoms [32]. These signs in FD may be directly related to increased lipid metabolism. The enzymes associated with the glycerophospholipid metabolic pathway are mainly phosphocholine transferase, phosphorylcholine-cytidyl-transferase, lecithin-cholesterol acyltransferase, and phosphatidylcholine transfer protein-β etc [33]. Therefore, phosphatidylcholine might participate in the development of FD through the following pathways: on the one hand, the metabolism of phosphatidylcholine is closely related to HDL (high density lipoprotein), which determines the maturity of HDL [34, 35]; on the other hand, phosphatidylcholine is balanced with phosphatidylinositol [36], while the latter is a precursor of inositol 1,4,5-triPhosphate (IP3)and diacyl glycerol (DG),and once the balance is broken, it will affect the normal functioning of various biological functions, such as cell secretion, muscle contraction, cell proliferation and differentiation [37–39]. In addition, the intermediate substance CDP-choline is closely related to the synthesis of phosphatidylcholine and has an important influence on the hypothalamic–pituitary–adrenal (HPA) axis [40–42], and as a psychosomatic disease, HPA axis disorders play an important role in the development of FD [43].

The present study has several limitations, in which no external sample set was used for validation, and there were uncertain causal relationships between the biomarkers and the occurrence of spleen deficiency syndrome. Although the combination of metabonomics data and clinical features did not show a good predictive function, to the best of our knowledge, this report describes the first human study investigating not only the clinical features but also metabolic changes of spleen deficiency-FD using an UPLC-Q-TOF-MS/MS based metabonomics approach. Future studies will focus on the combination of clinic characteristics and metabolic features, leading to a more complete understanding of FD with spleen deficiency syndrome. Additional research directed towards biological interpretation are expected regarding which biomarkers are related to the severity of clinical symptoms in FD and which metabolite levels are affected by drug intervention.

In conclusion, the clinical analysis revealed that decreased motilin was an independent risk factor in spleen deficiency-FD; furthermore, the serum metabonomics analysis yielded novel insights into small-molecule metabolic alterations in its progression, with 15 metabolites biomarkers continuously varying across the progression of FD, which could be applied as an additional diagnostic tool for detecting delayed gastric emptying of FD in practical clinic cases.

## Conclusions

This report describes the first human study investigating not only the clinical features but also metabolic changes of spleen deficiency-FD using an UPLC-Q-TOF-MS/MS based metabonomics approach, furthermore, this study provides supportive evidence that Spleen deficiency syndrome was associated with delayed gastric emptying and the glycerophospholipid metabolic pathway was perturbed in FD patients. The combination of metabolic biomarkers and clinical features provided us with new ideas for multidimensional diagnosis of FD.

## Additional files

**Additional file 1.** Minimum Standards of Reporting Checklist.

**Additional file 2.** Additional data.

## Abbreviations

FD: functional dyspepsia data
HC: healthy control
PDS: postprandial discomfort syndrome
UPLC-Q-TOF-MS/MS: ultra performance liquid chromatography quadrupole time-of-flight tandem mass spectrometry
GE: gastric emptying
AUC: area under the curve
PC: phosphatidyl choline
PE: phosphatidyl ethanolamine
PA: phosphatidic acid
PS: phosphatidyl serine
HDL: high density lipoprotein
IP3: inositol 1,4,5-triPhosphate
DG: diacyl glycerol
HPA: hypothalamic–pituitary–adrenal
