# Supplementary material for: Combination of 15 lipid metabolites and motilin to diagnose spleen-deficiency FD
Source: Chin Med. 2019 Apr 15;14:16. doi: 10.1186/s13020-019-0238-9 (PMC6466668; doi:10.1186/s13020-019-0238-9)
Supplement: Supplementary file 2 — Additional file 2. Additional data. [file 13020_2019_238_MOESM2_ESM.docx]

**Additional data**


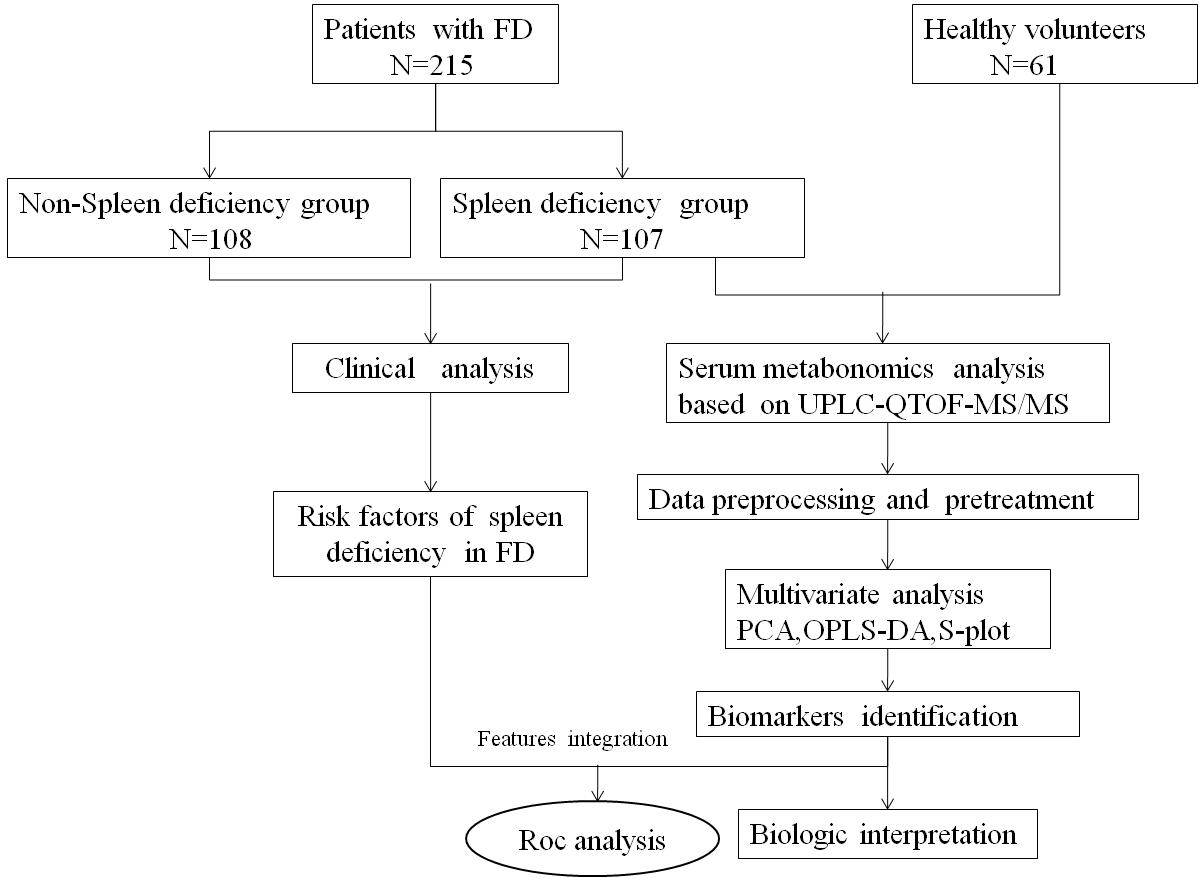


**Figure S1: An overview of workflow utilized in serum metabonomics analysis of FD.** This figure showed the process of recruiting patients of FD and screening biomarkers.

**Table 1S Rome III diagnostic criteria for functional dyspepsia**

| Functional dyspepsia | Subtype |
| --- | --- |
| The last 3 mo with symptom onset at least 6 mo before  diagnosis, and must include | Postprandial distress syndrome |
| 1 One or more of: | Must include one or both of the following: |
| a, Bothersome postprandial fullness | 1 Bothersome postprandial fullness, occurring after ordinary sized meals, at least several times per week |
| b, Early satiation | 2 Early satiation that prevents finishing a regular meal, at least several times per week Supportive criteria |
| c, Epigastric pain | 1 Upper abdominal bloating or postprandial nausea or excessive belching can be present |
| d, Epigastric burning  AND | 2 EPS may coexist |
| 2 No evidence of structural disease (including at upper  endoscopy) that is likely to explain the symptoms |  |

The Rome III diagnostic criteria for functional dyspepsia were detailed in this table, whichcwas the basis for our recruitment of patients.

**Table S2 Traditional Chinese medicine diagnostic criteria for spleen deficiency syndrome**

|  | Spleen-deficiency and Qi-stagnation |
| --- | --- |
| **Primary symptoms** | Abdominal distension, with obviously postprandial |
|  | Distress  Anorexia or little appetite |
| **Secondary symptoms** | Nausea; acid reflux/heartburn; loose stools; fatigue/  exhausted |
|  | Fat tongue with slightly white coating |

This table detailed the diagnostic criteria for TCM spleen deficiency syndrome.

**Table S3 Inclusion and exclusion criteria**

| Inclusion criteria  1 Age 18-70 yr, Chinese reading and writing ability  2 Meeting the Rome III diagnostic criteria for PDS  3 Having the symptom of “spleen-deficiency and Qi-stagnation”, “spleen-deficiency and damp-obstruction”, “spleen-Yang deficiency”.  4 Signing the informed consent form  Exclusion criteria  1 Combined with GI ulcer, erosive gastritis, atrophic gastritis, severe dysplasia of gastric mucosa or suspicious malignant lesion  2 Having overlap syndrome combined with gastroesophageal reflux disease or irritable bowel syndrome  3 Having alarm symptoms (weight loss, black or tar stool, dysphagia, *etc.*)  4 Having serious structural disease (disease of heart, lung, liver or kidney) or mental illness  5 History of surgery related with the gastrointestinal tract, except for appendectomy more than six months ago  6 Taking drugs which may affect the gastrointestinal tract, such as nonsteroidal anti-inflammatory drugs and aspirin  7 Allergy to the experimental medication  8 Difficulties in attending the trial (paralysis, serious mental illness, dementia, renal diseases, stroke, coronary atherosclerotic heart diseases, diabetes or  mental diseases, illiteracy, *etc.*)  9 Pregnant or breastfeeding  10 Refusing to sign the informed consent form |  |
| --- | --- |

This table detailed the inclusion and exclusion of patients with functional dyspepsia

**TableS4 Postprandial Discomfort Severity Scale**

| Syndrome | Severity | frequency |
| --- | --- | --- |
| **Postprandial fullness** | 0, absent;  1, mild(awareness of symptoms but easily tolerated); 2,moderate (interference with normal activities);  3, severe (incapacitating)Abdominal distension, with obviously postprandial | 0, absent (less than once per month);  1, rarely (less than once per week);  2, occasionally (less than three times per week); and 3, often (more than or three times per week) |
|  |  |  |
| **Early satiety** | 0, absent;  1, mild(awareness of symptoms but easily tolerated); 2,moderate (interference with normal activities);  3, severe (incapacitating)Abdominal distension, with obviously postprandial | 0, absent (less than once per month);  1, rarely (less than once per week);  2, occasionally (less than three times per week); and 3, often (more than or three times per week) |
| **Upper abdominal pain or discomfort** | 0, absent;  1, mild(awareness of symptoms but easily tolerated); 2,moderate (interference with normal activities);  3, severe (incapacitating)Abdominal distension, with obviously postprandial | 0, absent (less than once per month);  1, rarely (less than once per week);  2, occasionally (less than three times per week); and 3, often (more than or three times per week) |
| Heartburn | 0, absent;  1,mild(awareness of symptoms but easily tolerated); 2,moderate (interference with normal activities);  3,severe (incapacitating)Abdominal distension, with obviously postprandial | 0, absent (less than once per month);  1, rarely (less than once per week);  2, occasionally (less than three times per week); and 3, often (more than or three times per week) |

This table provided a score standard for postprandial discomfort based on the severity of the symptoms and the frequency of seizures.

**Table S5 Signature metabolites of spleen deficiency-FD group of XiYuan Hospital**

| **No** | **t_R_** | ***m/z*** | **Quasi-molecular ion** | **MS/MS** | **Formula** | **Name** | **VIP** |
| --- | --- | --- | --- | --- | --- | --- | --- |
| 1 | 6.29 | 496.3418 | [M+H]^+^ | 496.3407 | C_24_H_50_NO_7_P | LysoPC(16:0) | 25.4589 |
|  |  |  |  | 478.3301 |  |  |  |
|  |  |  |  | 240.0999 |  |  |  |
|  |  |  |  | 313.2740 |  |  |  |
|  |  |  |  | 184.0745 |  |  |  |
| 2 | 7.71 | 524.3720 | [M+H]^+^ | 524.3709 | C_26_H_54_NO_7_P | LysoPC(18:0) | 20.284 |
|  |  |  |  | 506.3611 |  |  |  |
|  |  |  |  | 341.3055 |  |  |  |
|  |  |  |  | 184.0742 |  |  |  |
|  |  |  |  | 104.1079 |  |  |  |
| 3 | 5.28 | 119.0862 | [M+H]^+^ | 119.0862 | C_9_H_10_ | alpha-Methylstyrene | 2.88241 |
|  |  |  |  | 91.0553 |  |  |  |
|  |  |  |  | 79.0563 |  |  |  |
| 4 | 4.02 | 318.3012 | [M+H]+ | 318.3014 | C_18_H_39_NO_3_ | Phytosphingosine | 2.66663 |
|  |  |  |  | 300.2928 |  |  |  |
| 5 | 5.87 | 568.3405 | [M+H]^+^ | 568.3395 | C_30_H_50_NO_7_P | PC(22:6/0:0) | 2.53445 |
|  |  |  |  | 550.3397 |  |  |  |
|  |  |  |  | 385.2707 |  |  |  |
|  |  |  |  | 258.1108 |  |  |  |
|  |  |  |  | 240.1007 |  |  |  |
| 6 | 4.78 | 302.3062 | [M+H]^+^ | 302.3067 | C_18_H_39_NO_2_ | Sphinganine | 2.49736 |
| 7 | 6.96 | 510.3564 | [M+H]^+^ | 510.3563 | C_25_H_52_NO_7_P | LysoPC(17:0) | 2.30291 |
|  |  |  |  | 492.3456 |  |  |  |
|  |  |  |  | 327.2885 |  |  |  |
|  |  |  |  | 184.0742 |  |  |  |
| 8 | 7.67 | 786.6001 | [M+H]^+^ | 786.5968 | C_44_H_84_NO_8_P | PC(18:2/18:0) | 2.05462 |
|  |  |  |  | 758.5676 |  |  |  |
|  |  |  |  | 506.3613 |  |  |  |
|  |  |  |  | 184.0744 |  |  |  |
| 9 | 3.65 | 316.2491 | [M+H]^+^ | 316.2494 | C_17_H_33_NO_4_ | L-Hexanoylcarnitine n-butyl ester | 1.92523 |
|  |  |  |  | 257.1758 |  |  |  |
|  |  |  |  | 144.1042 |  |  |  |
| 10 | 3.38 | 780.4917 | [M+H]^+^ | 780.4971 | C_42_H_70_NO_10_P | PS(14:0/22:6) | 1.44256 |
|  |  |  |  | 416.2158 |  |  |  |
| 11 | 5.89 | 564.3306 | [M+FA-H]^-^ | 564.3311 | C_26_H_50_NO_7_P | PC(18:2/0:0) | 17.4752 |
|  |  |  |  | 504.3101 |  |  |  |
|  |  |  |  | 279.2326 |  |  |  |
| 12 | 6.44 | 566.347 | [M+FA-H]^-^ | 566.3467 | C_26_H_52_NO_7_P | LysoPC(18:1) | 16.4113 |
|  |  |  |  | 506.3260 |  |  |  |
|  |  |  |  | 281.2486 |  |  |  |
| 13 | 5.93 | 588.3312 | [M+FA-H]^-^ | 588.3309 | C_28_H_50_NO_7_P | LysoPC(20:4) | 8.63692 |
|  |  |  |  | 528.3097 |  |  |  |
|  |  |  |  | 304.2356 |  |  |  |
| 14 | 5. 9 | 504.3102 | [M-H]^-^ | 504.3100 | C_25_H_48_NO_7_P | PC(17:2/0:0) | 7.75141 |
|  |  |  |  | 564.3310 |  |  |  |
|  |  |  |  | 224.0685 |  |  |  |
| 15 | 6.67 | 506.3259 | [M-H]^-^ | 506.3260 | C_25_H_50_NO_7_P | PC(17:1/0:0) | 6.86306 |
|  |  |  |  | 566.3467 |  |  |  |
|  |  |  |  | 224.0686 |  |  |  |
| 16 | 5.51 | 538.3150 | [M+FA-H]^-^ | 538.3140 | C_24_H_48_NO_7_P | LysoPC(16:1) | 5.26661 |
|  |  |  |  | 478.2940 |  |  |  |
|  |  |  |  | 253.2169 |  |  |  |
| 17 | 5.84 | 476.2783 | [M-H]^-^ | 476.2790 | C_23_H_44_NO_7_P | LysoPE(18:2/0:0) | 5.07686 |
|  |  |  |  | 279.2321 |  |  |  |
| 18 | 6.07 | 480.3100 | [M-H]^-^ | 480.3104 | C_23_H_48_NO_7_P | PC(15:0/0:0) | 4.65039 |
|  |  |  |  | 540.3307 |  |  |  |
|  |  |  |  | 241.0113 |  |  |  |
|  |  |  |  | 224.0672 |  |  |  |
| 19 | 7.65 | 480.3097 | [M-H]^-^ | 480.3099 | C_23_H_48_NO_7_P | LysoPE(0:0/18:0) | 4.48906 |
|  |  |  |  | 283.2633 |  |  |  |
| 20 | 6.98 | 554.3469 | [M+FA-H]^+^ | 554.3462 | C_25_H_52_NO_7_P | PC(16:0/O-1:0) | 4.06562 |
|  |  |  |  | 494.3450 |  |  |  |
|  |  |  |  | 269.2480 |  |  |  |
|  |  |  |  | 224.0686 |  |  |  |
| 21 | 7.35 | 433.2359 | [M-H]^-^ | 433.2357 | C_21_H_39_O_7_P | PA(18:2/0:0) | 3.7093 |
|  |  |  |  | 279.2325 |  |  |  |
| 22 | 6.25 | 452.2782 | [M-H]^-^ | 452.2784 | C_21_H_44_NO_7_P | LysoPE(0:0/16:0) | 3.3799 |
|  |  |  |  | 255.2326 |  |  |  |
| 23 | 9.89 | 685.4953 | [M+FA-H]^-^ | 279.2322 | C_41_H_68_O_5_ | DG(18:2/20:4/0:0) | 3.24488 |
|  |  |  |  | 303.2325 |  |  |  |
| 24 | 5.87 | 500.2784 | [M-H]^-^ | 500.2779 | C_25_H_44_NO_7_P | LysoPE(20:4/0:0) | 3.1548 |
|  |  |  |  | 303.2323 |  |  |  |
| 25 | 5.84 | 524.2784 | [M-H]^-^ | 524.2783 | C_27_H_44_NO_7_P | LysoPE(0:0/22:6) | 3.09952 |
|  |  |  |  | 327.2321 |  |  |  |
| 26 | 6.61 | 478.2939 | [M-H]^-^ | 478.2939 | C_23_H_46_NO_7_P | LysoPE(18:1/0:0) | 2.69198 |
|  |  |  |  | 281.2486 |  |  |  |
| 27 | 6.35 | 590.3465 | [M+FA-H]^-^ | 590.3456 | C_28_H_52_NO_7_P | LysoPC(20:3) | 2.20415 |
|  |  |  |  | 530.3234 |  |  |  |
|  |  |  |  | 305.2484 |  |  |  |
| 28 | 5.18 | 512.2991 | [M+FA-H]^-^ | 512.2988 | C_22_H_46_NO_7_P | LysoPC(14:0) | 2.14849 |
|  |  |  |  | 227.2009 |  |  |  |
| 29 | 7.03 | 592.3618 | [M+FA-H]^-^ | 592.3486 | C_28_H_54_NO_7_P | LysoPC(20:2) | 2.01523 |
|  |  |  |  | 307.3199 |  |  |  |
|  |  |  |  | 224.1376 |  |  |  |
|  |  |  |  | 241.2163 |  |  |  |

This table showed signature metabolites of spleen deficiency-FD group of XiYuan Hospital

**Table S6 Signature metabolites of spleen deficiency-FD group of GuangDong Province Traditional Chinese Medical Hospital**

| **No** | **t_R_** | ***m/z*** | **Quasi-molecular ion** | **MS/MS** | **Formula** | **Name** | **VIP** |
| --- | --- | --- | --- | --- | --- | --- | --- |
| 1 | 5.21 | 282.2805 | [M+H]^+^ | 282.2810 | C_18_H_35_NO | Elaidamide | 21.4962 |
|  |  |  |  | 264.2705 |  |  |  |
|  |  |  |  | 247.2440 |  |  |  |
|  |  |  |  | 97.1024 |  |  |  |
| 2 | 10.8 | 284.2961 | [M+H]^+^ | 284.2982 | C_18_H_37_NO | Stearamide | 15.2722 |
|  |  |  |  | 102.0912 |  |  |  |
|  |  |  |  | 83.0854 |  |  |  |
| 3 | 9.18 | 256.2648 | [M+H]^+^ | 256.2637 | C_16_H_33_NO | Palmitic amide | 14.264 |
|  |  |  |  | 172.1687 |  |  |  |
|  |  |  |  | 144.1381 |  |  |  |
|  |  |  |  | 130.1241 |  |  |  |
| 4 | 11.04 | 310.3112 | [M+H]^+^ | 240.2339 | C_20_H_39_NO | Oleoyl Ethyl Amide | 12.945 |
|  |  |  |  | 212.2019 |  |  |  |
|  |  |  |  | 198.1859 |  |  |  |
|  |  |  |  | 114.0923 |  |  |  |
| 5 | 6.31 | 540.3328 | [M+FA-H]^-^ | 540.3340 | C_24_H_50_NO_7_P | PC(16:0/0:0) | 22.6083 |
|  |  |  |  | 480.3281 |  |  |  |
|  |  |  |  | 255.2973 |  |  |  |
| 6 | 5.22 | 768.4745 | [M+FA-H]^-^ | 768.4728 | C_40_H_70_NO_8_P | PE(15:1/20:4) | 22.5158 |
|  |  |  |  | 722.4806 |  |  |  |
|  |  |  |  | 303.2899 |  |  |  |
|  |  |  |  | 239.1319 |  |  |  |
| 7 | 7.73 | 568.3608 | [M+FA-H]^-^ | 568.3580 | C_26_H_54_NO_7_P | LysoPC(18:0) | 17.0276 |
|  |  |  |  | 508.2527 |  |  |  |
|  |  |  |  | 283.3247 |  |  |  |
| 8 | 5.89 | 564.3305 | [M+FA-H]^-^ | 564.3281 | C_26_H_50_NO_7_P | LysoPC(18:2) | 15.751 |
|  |  |  |  | 504.3225 |  |  |  |
|  |  |  |  | 279.2937 |  |  |  |
| 9 | 6.67 | 566.3458 | [M+FA-H]^-^ | 566.3442 | C_26_H_52_NO_7_P | LysoPC(18:1) | 15.2272 |
|  |  |  |  | 506.3384 |  |  |  |
|  |  |  |  | 281.3098 |  |  |  |
| 10 | 9.74 | 745.4969 | [M+FA-H]^-^ | 745.4966 | C_39_H_73_O_8_P | PA(18:0/18:2) | 14.3854 |
|  |  |  |  | 279.2937 |  |  |  |
|  |  |  |  | 421.3631 |  |  |  |
| 11 | 6.29 | 480.312 | [M-H]^-^ | 480.3285 | C_23_H_48_NO_7_P | PC(15:0/0:0) | 12.0806 |
|  |  |  |  | 241.4475 |  |  |  |
|  |  |  |  | 224.1375 |  |  |  |
| 12 | 10.08 | 828.4954 | [M+FA-H]^-^ | 828.4946 | C_42_H_74_NO_10_P | PS(18:2/18:2) | 11.1989 |
|  |  |  |  | 768.4919 |  |  |  |
|  |  |  |  | 279.2935 |  |  |  |
| 13 | 5.22 | 785.4591 | [M+FA-H]^-^ | 785.4568 | C_40_H_69_O_10_P | PG(14:0/20:5) | 9.16012 |
|  |  |  |  | 770.4773 |  |  |  |
|  |  |  |  | 329.2837 |  |  |  |
|  |  |  |  | 227.2926 |  |  |  |
| 14 | 5.93 | 588.3286 | [M+FA-H]^-^ | 588.3215 | C_28_H_50_NO_7_P | LysoPC(20:4) | 8.42064 |
|  |  |  |  | 528.3161 |  |  |  |
|  |  |  |  | 304.2928 |  |  |  |
| 15 | 7.73 | 508.3434 | [M-H]^-^ | 508.3527 | C_25_H_52_NO_7_P | LysoPC(17:0) | 7.72844 |
|  |  |  |  | 224.1373 |  |  |  |
| 16 | 5.89 | 504.3109 | [M-H]^-^ | 504.3230 | C_25_H_48_NO_7_P | PC(17:2/0:0) | 7.50563 |
|  |  |  |  | 564.3283 |  |  |  |
|  |  |  |  | 224.1376 |  |  |  |
| 17 | 6.67 | 506.3276 | [M-H]^-^ | 506.3372 | C_25_H_50_NO_7_P | PC(17:1/0:0) | 6.72415 |
|  |  |  |  | 566.3422 |  |  |  |
|  |  |  |  | 224.1373 |  |  |  |
| 18 | 9.15 | 719.49 | [M+FA-H]^-^ | 719.4863 | C_37_H_71_O_8_P | PA(18:0/16:1) | 6.11691 |
|  |  |  |  | 659.4838 |  |  |  |
|  |  |  |  | 283.3218 |  |  |  |
|  |  |  |  | 253.2748 |  |  |  |
| 19 | 11.83 | 826.4805 | [M+FA-H]^-^ | 826.4777 | C_42_H_72_NO_10_P | PS(18:3/18:2) | 5.85827 |
|  |  |  |  | 766.4772 |  |  |  |
|  |  |  |  | 279.2932 |  |  |  |
|  |  |  |  | 277.2804 |  |  |  |
| 20 | 10.08 | 685.5261 | [M+FA-H]^-^ | 685.4916 | C_41_H_68_O_5_ | DG(18:2/20:4/0:0) | 5.66863 |
|  |  |  |  | 279.2928 |  |  |  |
|  |  |  |  | 303.2891 |  |  |  |
| 21 | 11.08 | 850.4733 | [M+FA-H]^-^ | 850.4739 | C_44_H_72_NO_10_P | PS(20:5/18:2) | 5.31529 |
|  |  |  |  | 790.4718 |  |  |  |
|  |  |  |  | 804.4962 |  |  |  |
|  |  |  |  | 279.2931 |  |  |  |
| 22 | 5.88 | 612.323 | [M+FA-H]^-^ | 612.3142 | C_30_H_50_NO_7_P | PC(22:6/0:0) | 4.40969 |
|  |  |  |  | 552.3096 |  |  |  |
|  |  |  |  | 327.2849 |  |  |  |
| 23 | 7.61 | 802.4888 | [M+FA-H]^-^ | 802.4863 | C_40_H_72_NO_10_P | PS(18:2/16:1) | 4.11822 |
|  |  |  |  | 742.4859 |  |  |  |
|  |  |  |  | 279.2937 |  |  |  |
| 24 | 10.55 | 594.3905 | [M+FA-H]^-^ | 594.3851 | C_28_H_56_NO_7_P | PC(18:1/2:0) | 4.08824 |
|  |  |  |  | 281.3079 |  |  |  |
| 25 | 9.7 | 773.5197 | [M+FA-H]^-^ | 773.5179 | C_43_H_83_O_9_P | SM(d18:2/18:0) | 4.02918 |
|  |  |  |  | 804.4944 |  |  |  |
|  |  |  |  | 279.2933 |  |  |  |
|  |  |  |  | 742.4874 |  |  |  |
|  |  |  |  | 279.2930 |  |  |  |
| 26 | 5.84 | 524.2815 | [M-H]^-^ | 524.2858 | C_27_H_44_NO_7_P | LysoPE(0:0/22:6) | 3.74088 |
|  |  |  |  | 327.2850 |  |  |  |
| 27 | 5.51 | 538.3165 | [M+FA-H]^-^ | 538.3192 | C_24_H_48_NO_7_P | LysoPC(16:1) | 3.6254 |
|  |  |  |  | 478.3126 |  |  |  |
|  |  |  |  | 253.2822 |  |  |  |
| 28 | 8.93 | 747.5132 | [M+FA-H]^-^ | 747.5109 | C_39_H_75_O_8_P | PA(20:1/16:0) | 3.46395 |
|  |  |  |  | 687.5060 |  |  |  |
|  |  |  |  | 281.3091 |  |  |  |
|  |  |  |  | 283.3185 |  |  |  |
| 29 | 5.93 | 528.3125 | [M-H]^-^ | 528.3158 | C_27_H_48_NO_7_P | LysoPE(0:0/22:4) | 3.46085 |
|  |  |  |  | 514.1483 |  |  |  |
|  |  |  |  | 331.2910 |  |  |  |
| 30 | 10.08 | 742.4886 | [M+FA-H]^-^ | 742.4855 | C_39_H_72_NO_7_P | PE(18:3)/P-16:0) | 3.0823 |
|  |  |  |  | 255.2976 |  |  |  |
| 31 | 5.84 | 476.2784 | [M-H]^-^ | 476.2975 | C_23_H_44_NO_7_P | LysoPE(18:2/0:0) | 2.99044 |
|  |  |  |  | 279.2932 |  |  |  |
| 32 | 6.16 | 619.2879 | [M-H]^-^ | 619.2714 | C_29_H_49_O_12_P | Arachidonoylglycerophosphoinositol | 2.94028 |
|  |  |  |  | 303.2896 |  |  |  |
| 33 | 6.35 | 590.344 | [M+FA-H]^-^ | 590.3359 | C_28_H_52_NO_7_P | LysoPC(20:3) | 2.73133 |
|  |  |  |  | 530.3290 |  |  |  |
|  |  |  |  | 305.3050 |  |  |  |
| 34 | 6.25 | 452.2796 | [M-H]^+^ | 452.3015 | C_21_H_44_NO_7_P | LysoPE(0:0/16:0) | 2.70168 |
|  |  |  |  | 255.2975 |  |  |  |
| 35 | 5.87 | 500.2795 | [M-H]^-^ | 500.2914 | C_25_H_44_NO_7_P | LysoPE(20:4/0:0) | 2.69128 |
|  |  |  |  | 303.2889 |  |  |  |
| 36 | 7.61 | 802.5607 | [M+FA-H]^-^ | 802.4868 | C_42_H_80_NO_8_P | PC(18:2/16:0) | 2.47169 |
|  |  |  |  | 742.4865 |  |  |  |
|  |  |  |  | 253.1688 |  |  |  |
|  |  |  |  | 281.3084 |  |  |  |
| 37 | 6.98 | 554.3467 | [M+FA-H]^-^ | 554.3475 | C_25_H_52_NO_7_P | PC(16:0/O-1:0) | 2.23254 |
|  |  |  |  | 494.3412 |  |  |  |
|  |  |  |  | 269.3105 |  |  |  |
|  |  |  |  | 224.1371 |  |  |  |

This table shows signature metabolites of spleen deficiency-FD group of GuangDong Province Traditional Chinese Medical Hospital

**Table S7 Signature metabolites of spleen deficiency-FD group of Wuhan Integrated TCM &WESTERN Medicine Hospital**

| **No** | **t_R_** | ***m/z*** | **Quasi-molecular ion** | **MS/MS** | **Formula** | **Name** | **VIP** |
| --- | --- | --- | --- | --- | --- | --- | --- |
| 1 | 5.21 | 282.2805 | [M+H]^+^ | 282.2810 | C_18_H_35_NO | Elaidamide | 21.4962 |
|  |  |  |  | 264.2705 |  |  |  |
|  |  |  |  | 247.2440 |  |  |  |
|  |  |  |  | 97.1024 |  |  |  |
| 2 | 10.8 | 284.2961 | [M+H]^+^ | 284.2982 | C_18_H_37_NO | Stearamide | 15.2722 |
|  |  |  |  | 102.0912 |  |  |  |
|  |  |  |  | 83.0854 |  |  |  |
| 3 | 9.18 | 256.2648 | [M+H]^+^ | 256.2637 | C_16_H_33_NO | Palmitic amide | 14.264 |
|  |  |  |  | 172.1687 |  |  |  |
|  |  |  |  | 144.1381 |  |  |  |
|  |  |  |  | 130.1241 |  |  |  |
| 4 | 11.04 | 310.3112 | [M+H]^+^ | 240.2339 | C_20_H_39_NO | Oleoyl Ethyl Amide | 12.945 |
|  |  |  |  | 212.2019 |  |  |  |
|  |  |  |  | 198.1859 |  |  |  |
|  |  |  |  | 114.0923 |  |  |  |
| 5 | 6.31 | 540.3328 | [M+FA-H]^-^ | 540.3340 | C_24_H_50_NO_7_P | PC(16:0/0:0) | 22.6083 |
|  |  |  |  | 480.3281 |  |  |  |
|  |  |  |  | 255.2973 |  |  |  |
| 6 | 5.22 | 768.4745 | [M+FA-H]^-^ | 768.4728 | C_40_H_70_NO_8_P | PE(15:1/20:4) | 22.5158 |
|  |  |  |  | 722.4806 |  |  |  |
|  |  |  |  | 303.2899 |  |  |  |
|  |  |  |  | 239.1319 |  |  |  |
| 7 | 7.73 | 568.3608 | [M+FA-H]^-^ | 568.3580 | C_26_H_54_NO_7_P | LysoPC(18:0) | 17.0276 |
|  |  |  |  | 508.2527 |  |  |  |
|  |  |  |  | 283.3247 |  |  |  |
| 8 | 5.89 | 564.3305 | [M+FA-H]^-^ | 564.3281 | C_26_H_50_NO_7_P | LysoPC(18:2) | 15.751 |
|  |  |  |  | 504.3225 |  |  |  |
|  |  |  |  | 279.2937 |  |  |  |
| 9 | 6.67 | 566.3458 | [M+FA-H]^-^ | 566.3442 | C_26_H_52_NO_7_P | LysoPC(18:1) | 15.2272 |
|  |  |  |  | 506.3384 |  |  |  |
|  |  |  |  | 281.3098 |  |  |  |
| 10 | 9.74 | 745.4969 | [M+FA-H]^-^ | 745.4966 | C_39_H_73_O_8_P | PA(18:0/18:2) | 14.3854 |
|  |  |  |  | 279.2937 |  |  |  |
|  |  |  |  | 421.3631 |  |  |  |
| 11 | 6.29 | 480.312 | [M-H]^-^ | 480.3285 | C_23_H_48_NO_7_P | PC(15:0/0:0) | 12.0806 |
|  |  |  |  | 241.4475 |  |  |  |
|  |  |  |  | 224.1375 |  |  |  |
| 12 | 10.08 | 828.4954 | [M+FA-H]^-^ | 828.4946 | C_42_H_74_NO_10_P | PS(18:2/18:2) | 11.1989 |
|  |  |  |  | 768.4919 |  |  |  |
|  |  |  |  | 279.2935 |  |  |  |
| 13 | 5.22 | 785.4591 | [M+FA-H]^-^ | 785.4568 | C_40_H_69_O_10_P | PG(14:0/20:5) | 9.16012 |
|  |  |  |  | 770.4773 |  |  |  |
|  |  |  |  | 329.2837 |  |  |  |
|  |  |  |  | 227.2926 |  |  |  |
| 14 | 5.93 | 588.3286 | [M+FA-H]^-^ | 588.3215 | C_28_H_50_NO_7_P | LysoPC(20:4) | 8.42064 |
|  |  |  |  | 528.3161 |  |  |  |
|  |  |  |  | 304.2928 |  |  |  |
| 15 | 7.73 | 508.3434 | [M-H]^-^ | 508.3527 | C_25_H_52_NO_7_P | LysoPC(17:0) | 7.72844 |
|  |  |  |  | 224.1373 |  |  |  |
| 16 | 5.89 | 504.3109 | [M-H]^-^ | 504.3230 | C_25_H_48_NO_7_P | PC(17:2/0:0) | 7.50563 |
|  |  |  |  | 564.3283 |  |  |  |
|  |  |  |  | 224.1376 |  |  |  |
| 17 | 6.67 | 506.3276 | [M-H]^-^ | 506.3372 | C_25_H_50_NO_7_P | PC(17:1/0:0) | 6.72415 |
|  |  |  |  | 566.3422 |  |  |  |
|  |  |  |  | 224.1373 |  |  |  |
| 18 | 9.15 | 719.49 | [M+FA-H]^-^ | 719.4863 | C_37_H_71_O_8_P | PA(18:0/16:1) | 6.11691 |
|  |  |  |  | 659.4838 |  |  |  |
|  |  |  |  | 283.3218 |  |  |  |
|  |  |  |  | 253.2748 |  |  |  |
| 19 | 11.83 | 826.4805 | [M+FA-H]^-^ | 826.4777 | C_42_H_72_NO_10_P | PS(18:3/18:2) | 5.85827 |
|  |  |  |  | 766.4772 |  |  |  |
|  |  |  |  | 279.2932 |  |  |  |
|  |  |  |  | 277.2804 |  |  |  |
| 20 | 10.08 | 685.5261 | [M+FA-H]^-^ | 685.4916 | C_41_H_68_O_5_ | DG(18:2/20:4/0:0) | 5.66863 |
|  |  |  |  | 279.2928 |  |  |  |
|  |  |  |  | 303.2891 |  |  |  |
| 21 | 11.08 | 850.4733 | [M+FA-H]^-^ | 850.4739 | C_44_H_72_NO_10_P | PS(20:5/18:2) | 5.31529 |
|  |  |  |  | 790.4718 |  |  |  |
|  |  |  |  | 804.4962 |  |  |  |
|  |  |  |  | 279.2931 |  |  |  |
| 22 | 5.88 | 612.323 | [M+FA-H]^-^ | 612.3142 | C_30_H_50_NO_7_P | PC(22:6/0:0) | 4.40969 |
|  |  |  |  | 552.3096 |  |  |  |
|  |  |  |  | 327.2849 |  |  |  |
| 23 | 7.61 | 802.4888 | [M+FA-H]^-^ | 802.4863 | C_40_H_72_NO_10_P | PS(18:2/16:1) | 4.11822 |
|  |  |  |  | 742.4859 |  |  |  |
|  |  |  |  | 279.2937 |  |  |  |
| 24 | 10.55 | 594.3905 | [M+FA-H]^-^ | 594.3851 | C_28_H_56_NO_7_P | PC(18:1/2:0) | 4.08824 |
|  |  |  |  | 281.3079 |  |  |  |
| 25 | 9.7 | 773.5197 | [M+FA-H]^-^ | 773.5179 | C_43_H_83_O_9_P | SM(d18:2/18:0) | 4.02918 |
|  |  |  |  | 804.4944 |  |  |  |
|  |  |  |  | 279.2933 |  |  |  |
| 26 | 7.61 | 802.4883 | [M+FA-H]^-^ | 802.4828 | C_40_H_72_NO_10_P | PS(18:2/16:1) | 3.94547 |
|  |  |  |  | 742.4874 |  |  |  |
|  |  |  |  | 279.2930 |  |  |  |
| 27 | 5.84 | 524.2815 | [M-H]^-^ | 524.2858 | C_27_H_44_NO_7_P | LysoPE(0:0/22:6) | 3.74088 |
|  |  |  |  | 327.2850 |  |  |  |
| 28 | 5.51 | 538.3165 | [M+FA-H]^-^ | 538.3192 | C_24_H_48_NO_7_P | LysoPC(16:1) | 3.6254 |
|  |  |  |  | 478.3126 |  |  |  |
|  |  |  |  | 253.2822 |  |  |  |
| 29 | 8.93 | 747.5132 | [M+FA-H]^-^ | 747.5109 | C_39_H_75_O_8_P | PA(20:1/16:0) | 3.46395 |
|  |  |  |  | 687.5060 |  |  |  |
|  |  |  |  | 281.3091 |  |  |  |
|  |  |  |  | 283.3185 |  |  |  |
| 30 | 5.93 | 528.3125 | [M-H]^-^ | 528.3158 | C_27_H_48_NO_7_P | LysoPE(0:0/22:4) | 3.46085 |
|  |  |  |  | 514.1483 |  |  |  |
|  |  |  |  | 331.2910 |  |  |  |
| 31 | 10.08 | 742.4886 | [M+FA-H]^-^ | 742.4855 | C_39_H_72_NO_7_P | PE(18:3)/P-16:0) | 3.0823 |
|  |  |  |  | 255.2976 |  |  |  |
| 32 | 5.84 | 476.2784 | [M-H]^-^ | 476.2975 | C_23_H_44_NO_7_P | LysoPE(18:2/0:0) | 2.99044 |
|  |  |  |  | 279.2932 |  |  |  |
| 33 | 6.16 | 619.2879 | [M-H]^-^ | 619.2714 | C_29_H_49_O_12_P | Arachidonoylglycerophosphoinositol | 2.94028 |
|  |  |  |  | 303.2896 |  |  |  |
| 34 | 6.35 | 590.344 | [M+FA-H]^-^ | 590.3359 | C_28_H_52_NO_7_P | LysoPC(20:3) | 2.73133 |
|  |  |  |  | 530.3290 |  |  |  |
|  |  |  |  | 305.3050 |  |  |  |
| 35 | 6.25 | 452.2796 | [M-H]^+^ | 452.3015 | C_21_H_44_NO_7_P | LysoPE(0:0/16:0) | 2.70168 |
|  |  |  |  | 255.2975 |  |  |  |
| 36 | 5.87 | 500.2795 | [M-H]^-^ | 500.2914 | C_25_H_44_NO_7_P | LysoPE(20:4/0:0) | 2.69128 |
|  |  |  |  | 303.2889 |  |  |  |
| 37 | 7.61 | 802.5607 | [M+FA-H]^-^ | 802.4868 | C_42_H_80_NO_8_P | PC(18:2/16:0) | 2.47169 |
|  |  |  |  | 742.4865 |  |  |  |
|  |  |  |  | 253.1688 |  |  |  |
|  |  |  |  | 281.3084 |  |  |  |
| 38 | 6.98 | 554.3467 | [M+FA-H]^-^ | 554.3475 | C_25_H_52_NO_7_P | PC(16:0/O-1:0) | 2.23254 |
|  |  |  |  | 494.3412 |  |  |  |
|  |  |  |  | 269.3105 |  |  |  |
|  |  |  |  | 224.1371 |  |  |  |

This table showed signature metabolites of spleen deficiency-FD group of Wuhan Integrated TCM &WESTERN Medicine Hospital
